# Predicting abiotic stress-responsive miRNA in plants based on multi-source features fusion and graph neural network

**DOI:** 10.1186/s13007-024-01158-7

**Published:** 2024-02-24

**Authors:** Liming Chang, Xiu Jin, Yuan Rao, Xiaodan Zhang

**Affiliations:** 1 College of Information and Artificial Intelligence, Anhui Agricultural University, Hefei, 230036 China; 2 Anhui Province Key Laboratory of Smart Agricultural Technology and Equipment, Anhui Agricultural University, Hefei, 230036 China

**Keywords:** miRNA-abiotic stress association, Graph neural network, Multi-source features, Graph autoencoder

## Abstract

**Background:**

More and more studies show that miRNA plays a crucial role in plants' response to different abiotic stresses. However, traditional experimental methods are often expensive and inefficient, so it is important to develop efficient and economical computational methods. Although researchers have developed machine learning-based method, the information of miRNAs and abiotic stresses has not been fully exploited. Therefore, we propose a novel approach based on graph neural networks for predicting potential miRNA-abiotic stress associations.

**Results:**

In this study, we fully considered the multi-source feature information from miRNAs and abiotic stresses, and calculated and integrated the similarity network of miRNA and abiotic stress from different feature perspectives using multiple similarity measures. Then, the above multi-source similarity network and association information between miRNAs and abiotic stresses are effectively fused through heterogeneous networks. Subsequently, the Restart Random Walk (RWR) algorithm is employed to extract global structural information from heterogeneous networks, providing feature vectors for miRNA and abiotic stress. After that, we utilized the graph autoencoder based on GIN (Graph Isomorphism Networks) to learn and reconstruct a miRNA-abiotic stress association matrix to obtain potential miRNA-abiotic stress associations. The experimental results show that our model is superior to all known methods in predicting potential miRNA-abiotic stress associations, and the AUPR and AUC metrics of our model achieve 98.24% and 97.43%, respectively, under five-fold cross-validation.

**Conclusions:**

The robustness and effectiveness of our proposed model position it as a valuable approach for advancing the field of miRNA-abiotic stress association prediction.

## Key points

We propose an innovative approach based on multi-source similarity network fusion and graph autoencoder for predicting potential miRNA-abiotic stress associations.

We pioneer the application of graph neural networks in predicting miRNA-abiotic stress associations, achieving more accurate predictive performance than all known methods.

We also introduce a machine learning model based on multi-source similarity network fusion, showcasing its superiority over existing machine learning-based models.

## Background

MicroRNAs (miRNAs) are naturally occurring non-coding molecules comprising endogenous single-stranded RNAs, typically ranging from 21 to 25 nucleotides in length. They are ubiquitous in diverse organisms, including animals, green algae, plants, and viruses [1, 2]. miRNAs play a pivotal role in various fundamental biological processes [3], encompassing cell differentiation, development, cell cycle, apoptosis, and more [2, 4, 5]. Extensive research has validated that miRNAs significantly regulate genes, exerting their biological functions by inhibiting or degrading mRNA post-transcription [2].

Moreover, a growing body of evidence underscores the critical involvement of miRNAs in the plant's response to various abiotic stresses, enabling plants to adapt effectively [6–9]. For instance, Zhou et al. utilized a microarray platform to conduct comprehensive genome-wide profiling and analysis of miRNAs during different stages of rice development under drought stress. They observed significant down-regulation of 11 miRNAs and up-regulation of 8 miRNAs in response to drought stress [10].

Researchers have identified miRNAs responsive to a range of abiotic stresses such as drought [10–17], cold [18–21], heat [22–24], light [25–31], salt [17, 32, 33], and oxidative stress [34–38] in various crop species. Wang et al. [39], for example, discovered that miR398 expression was significantly suppressed under salt stress in cotton. Xie et al. [40] reported down-regulation of miR408 in cotton under drought stress using deep sequencing. Additionally, in Arabidopsis thaliana, miR169 expression was induced under salt stress [41], while miR398 expression was induced by UVB light but inhibited under salt, cold, and oxidative stress [35, 42].

The aforementioned studies collectively underscore the critical role of miRNAs in plant responses to diverse abiotic stresses. Identifying stress-responsive miRNAs in crops holds significant potential for developing stress-resistant varieties. Furthermore, comprehending the intricate interplay between miRNAs and abiotic stress is vital for understanding how organisms respond to environmental changes. Hence, employing suitable experimental or computational methodologies to investigate the miRNA-abiotic stress associations is imperative.

Traditional experimental approaches for identifying potential miRNA-abiotic stress associations primarily involve RT-PCR, cloning, RNA microarrays, northern blots, next-generation sequencing (NGS), and deep sequencing technologies [43–45]. Additionally, several authoritative bioinformatics databases, such as PncStress [46], PAS-miR [47], have been established to store miRNA-abiotic stress associations obtained through wet lab experiments and sequencing methods. However, these experimental and high-throughput sequencing techniques necessitate substantial financial investments and computational resources, rendering them less efficient. Therefore, there is an urgent need to develop efficient and cost-effective computational methods to predict potential miRNA-abiotic stress associations.

The machine learning-based approach represents a prevalent computational method for predicting such associations. Over the years, researchers have conducted extensive research on this front. For instance, Meher et al. [48] developed ASRmiRNA, a machine learning-based prediction tool, which employs the PseKNC [49] method to extract features from miRNA sequences. Subsequently, Support Vector Machines (SVM) are utilized to predict potential miRNA-abiotic stress associations, leveraging the obtained feature representations.

However, ASRmiRNA possesses certain limitations. Notably, it overlooks the contribution of abiotic stress information during association prediction, focusing solely on miRNA sequence information. This limitation hampers its predictive performance. Additionally, ASRmiRNA treats the prediction as a binary classification problem, providing no insight into the specific abiotic stress associated with a particular miRNA. Thus, a more precise prediction regarding the miRNA-abiotic stress association remains elusive.

In recent years, Graph Neural Networks (GNNs) have gained prominence in bioinformatics, exhibiting exceptional performance in association prediction problems. GNNs excel at learning topological information within graph structures, making them particularly effective for association prediction. For instance, Wang et al. [50] proposed an algorithm based on Graph Convolutional Networks (GCN) to predict circRNA-disease associations. This algorithm utilizes FastGCN and the Penalty Attribute Forest (Forest PA) algorithm to predict potential associations between circRNA and disease. Li et al. [51] proposed a computational model called DeepCMI based on circRNA miRNA biomedical maps to predict potential circRNA miRNA associations. Wang et al. [52] proposed a computational method KGDCMI based on multi-source information extraction and fusion to predict the interaction between circRNA and miRNA. Li et al. [53] propose the PPAEDTI model, which uses the graph personalized propagation technique to predict drug-target interactions from the known interaction network. Similarly, other models employing GNNs have made significant strides in association prediction problems [53–55].

To overcome the limitations of ASRmiRNA and capitalize on the success of GNNs in association prediction, we propose a novel method based on the fusion of multi-source similarity networks and graph autoencoder for predicting potential miRNA-abiotic stress associations. Our approach involves collecting and processing miRNA-abiotic stress association data from the PncStress database, constructing the miRNA-abiotic stress association matrix, and considering multi-source feature information from miRNA and abiotic stress. The method integrates similarity networks from various perspectives, combining them to create the final miRNA-abiotic stress heterogeneous network. Subsequently, the RWR algorithm is employed to extract global structural information from heterogeneous networks, providing feature vectors for miRNA and abiotic stress. These feature representations facilitate the prediction of potential miRNA-abiotic stress associations using an encoder-decoder model built upon the GIN model. The model exhibits superior performance compared to traditional machine learning models and commonly used graph neural network models, making it a promising approach for precise miRNA-abiotic stress association prediction.

In summary, our contributions encompass proposing an innovative approach based on multi-source similarity network fusion and graph autoencoder for predicting potential miRNA-abiotic stress associations. Our method comprehensively considers multi-source feature information from miRNA and abiotic stress, leveraging various similarity networks to enhance predictive performance. We also introduce a machine learning model based on multi-source similarity network fusion, showcasing its superiority over existing machine learning-based models. Furthermore, our study pioneers the application of graph neural networks in predicting miRNA-abiotic stress associations, achieving more accurate predictive performance compared to traditional machine learning models and commonly used graph neural network models. The robustness and effectiveness of our proposed model position it as a valuable approach for advancing the field of miRNA-abiotic stress association prediction.

## Materials and methods

### Overview

**Fig. 1:**
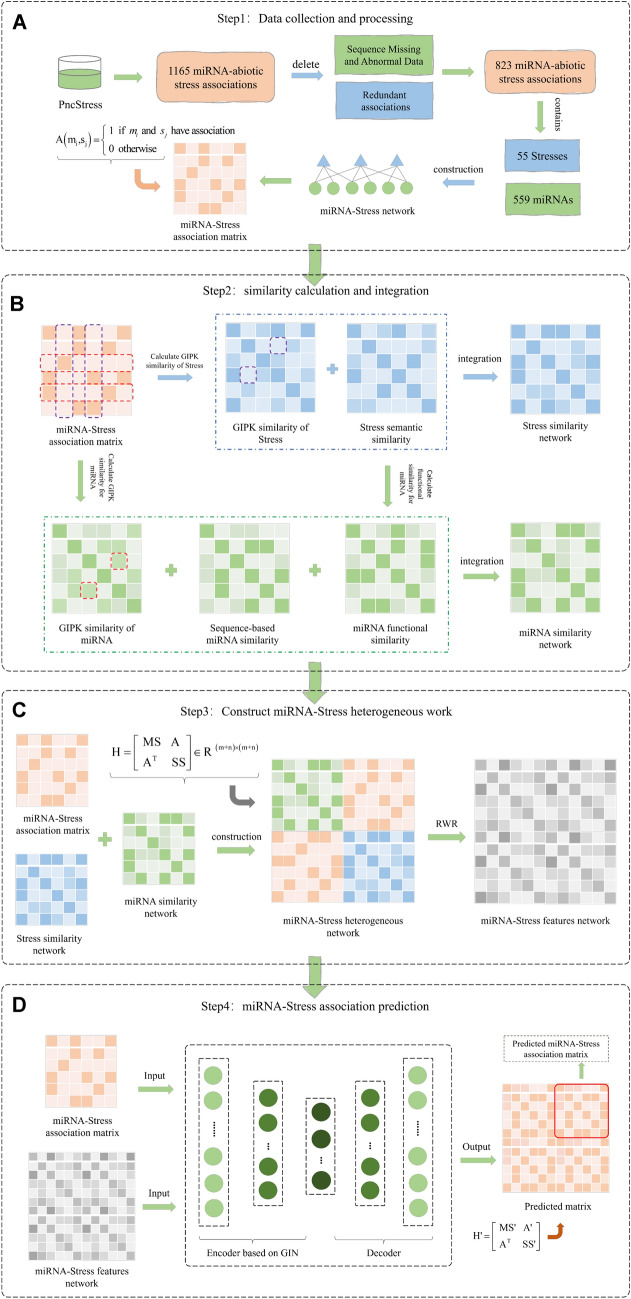
The workflow of our proposed model is delineated as follows: **A** Data Collection and Processing: We commence by gathering and meticulously processing miRNA-abiotic stress associations from the PncStress database, utilizing this curated dataset to construct the miRNA-abiotic stress association matrix. **B** Similarity Calculation and Integration: Leveraging the multi-source feature information in miRNA and abiotic stress, we employ various similarity measures to compute multiple similarity networks. These networks are then amalgamated to form an integrated miRNA and abiotic stress similarity network. **C** Constructing the miRNA-abiotic stress Heterogeneous Network: Next, we amalgamate the integrated miRNA similarity network, the integrated abiotic stress similarity network, and the miRNA-abiotic stress association network, culminating in the creation of a comprehensive miRNA-abiotic stress heterogeneous network. Subsequently, the RWR is deployed to glean meaningful node representations within the network. **D** miRNA-Abiotic Stress Association Prediction: In this crucial step, our model embarks on learning and reconstructing the miRNA-abiotic stress association network through the encoding and decoding processes. This iterative reconstruction enables us to deduce potential miRNA-abiotic stress associations with precision

In this research, we present a novel model rooted in the fusion of multi-source similarity network and graph autoencoder, aimed at predicting potential associations between miRNAs and abiotic stresses. The overarching model framework, as illustrated in Fig. 1, encompasses four fundamental modules: data collection and processing, similarity calculation and integration, constructing the miRNA-abiotic stress heterogeneous network, and miRNA-abiotic stress association prediction.

In the initial module, which focused on data collection and preprocessing, we gathered established miRNA-abiotic stress associations from the PncStress database [46]. Following meticulous processing, a total of 823 miRNA-abiotic stress association pairs were obtained, forming the basis for constructing the miRNA-abiotic stress association matrix.

The subsequent module, concerning similarity calculation and integration, fully accounts for multi-source features of miRNAs and abiotic stress factors. We employed various similarity measures to calculate the similarity network for miRNA and abiotic stress from multiple perspectives, to comprehensively characterize the information of miRNA and biotic stress. For miRNAs, this involved considering sequence similarity, functional similarity, and Gaussian interaction profile kernel (GIPK) similarity. Similarly, abiotic stress data was analyzed for semantic similarity and GIPK similarity. Following this, the disparate sources of similarity data were harmonized to derive the ultimate miRNA similarity network and abiotic stress similarity network.

In the module of constructing miRNA-abiotic stress heterogeneous networks, we combined the integrated miRNA similarity network, the integrated abiotic stress similarity network, and the miRNA-abiotic stress association network to formulate a comprehensive heterogeneous network. To capture the network's global structure, the RWR algorithm was employed. The RWR algorithm yielded steady-state matrices, which, in turn, facilitated the characterization of miRNA and abiotic stress nodes as feature vectors. Consequently, feature representations for miRNAs and abiotic stress were acquired.

Finally, in the miRNA-abiotic stress association prediction module, our model was deployed to predict potential associations. The model is constituted of an encoder and decoder, with the encoder grounded in the GIN model. GIN is instrumental in effectively extracting the implicit topological properties within the graph and acquiring an efficient representation of the graph's structure. It employs a neighbor aggregation strategy, iteratively updating feature vectors of specific nodes by aggregating those of their neighbors. Subsequently, after multiple iterations, the encoder's feature vectors were employed in reconstructing the miRNA-abiotic stress association matrix. This reconstruction formed the basis for predicting potential miRNA-abiotic stress associations through our model.

### miRNA-abiotic stress associations

We curated a total of 1165 established miRNA-abiotic stress associations sourced from the PncStress database [46], which are succinctly summarized in Table 1. The database encompasses 4227 experimentally validated associations involving various non-coding RNAs, including miRNAs, LncRNAs, and circRNAs, across 114 distinct plant species and in response to 48 biotic and 91 abiotic stresses. We removed redundant associations and abnormal data with missing miRNA sequences from the obtained miRNA-abiotic stress association data based on the principles of data preprocessing.

**Table 1:** Summary of data

	miRNA	Stress	Known associations
PncStress	721	55	1165
Used	559	55	823

After processing, we refined the dataset to comprise 823 miRNA-abiotic stress associations, encompassing 559 unique miRNAs and 55 distinct abiotic stresses. We further analyzed the composition of the dataset based on the number of associations under each species, as shown in Table 2. To obtain negative samples, we used the method proposed by Li et al. [51] in which negative samples were randomly sampled from the unlabeled samples.

**Table 2:** The distribution of association numbers under each species

Species	Associations	Species	Associations	Species	Associations
*Oryza sativa*	189	*Nicotiana tabacum*	62	*Brassica napus*	41
*Arabidopsis thaliana*	81	*Zea mays*	49	*Solanum lycopersicum*	34
*Populus trichocarpa*	78	*Glycine max*	49	*Triticum aestivum*	30
*Linum usitatissimum*	24	*Medicago truncatula*	21	*Phaseolus vulgaris*	13
*Physcomitrella patens*	23	*Gossypium hirsutum*	20	*Brassica rapa*	10
*Prunus persica*	22	*Hordeum vulgare*	13	*Brachypodium distachyon*	7
*Saccharum spp*	6	*Ricinus communis*	4	*Sorghum bicolor*	3
*Oryza rufipogon*	5	*Rehmannia glutinosa*	3	*Malus domestica*	2
*Glycine soja*	4	*Festuca arundinacea*	3	*Brassica campestris*	1
*Brassica juncea*	1				
Total	823				

We opted to employ an adjacency matrix to encapsulate the known miRNA-abiotic stress associations. In this matrix representation, denoted as ${\text{A}} \in {\text{R}}^{{\text{m }} \times {\text{ n}}}$, m signifies the number of miRNAs, and n signifies the number of abiotic stresses. The value at position ${\text{A}}_{{\text{ij}}}$ is assigned as 1 when miRNA ${\text{m}}_{\text{i}}$ correlates with abiotic stress ${\text{s}}_{\text{j}}$, conversely, it is set to 0 if there is no association between the respective miRNA and abiotic stress. The adjacency matrix ${\text{A}} \in {\text{R}}^{{\text{m }} \times {\text{ n}}}$ is described as:

$${\text{A}}\left( {{\text{m}}_{\text{i}} {\text{,s}}_{\text{j}} } \right) = \left\{ \begin{gathered} {\text{ 1 if }}m_i {\text{ and }}s_j {\text{ have association}} \hfill \\ {\text{ 0\;otherwise}} \hfill \\ \end{gathered} \right.$$

### Similarity calculation and integration

#### Abiotic stress semantic similarity

The paramount objective in calculating the semantic similarity of abiotic stress lies in acquiring an effective vector representation for each abiotic stress. To this end, we introduce the word2vec algorithm [56], a neural network-based word embedding technique renowned for its ability to map words into a high-dimensional vector space. This mapping ensures that words sharing similar semantics are situated in closer proximity within the vector space. Specifically, Word2vec is a method used to assign a fixed-length real value vector $V(m) \in R^m$ to any word $w$ in a dictionary $D$, where $V(m)$ represents the word vector of $w$ and $m$ is the length of the word vector. The collection of these vectors forms a word vector space, with each vector being considered as a point in space. The lexical or semantic similarity between words can be determined by measuring the distance between their respective points.

Word2vec is widely adopted in the realm of natural language processing, including recommendation systems [57], machine translation [58], semantic similarity computation [59], and text classification [60], consistently delivering noteworthy outcomes. In the field of association prediction, word2vec is also widely used. For example, Przybyszewski et al. [61]. applied word2vec to predict the associations between miRNA and target, using the word2vec method to accurately encode RNA sequence information, combined with a graph neural network for classification, and achieved good prediction results. Zhang et al. [62]. predicted the associations between Drug and Target by using word2vec to represent the potential features of small compounds and target proteins.

In our experiment, we utilize the word2vec algorithm to compute the semantic similarity among abiotic stresses. Specifically, we initially applied the word2vec algorithm to obtain an effective vector representation for each abiotic stress. In this context, we configure the vector dimension to be 100, thereby representing each abiotic stress as a vector of size 1 × 100. Subsequently, upon obtaining the vector representations, we employ the cosine similarity metric to quantify the similarity between abiotic stresses.

The cosine similarity metric yields values within the range of − 1 to 1, where a value approaching 1 signifies a higher degree of similarity between the two abiotic stresses. Conversely, a value of 0 implies a lack of significant similarity between the two stresses. The calculation of cosine similarity is elucidated by the following formula:

$$Sem\left( {s_i ,s_j } \right) = \frac{{vector\left( {s_i } \right) \cdot vector\left( {s_j } \right)}}{{\left\| {vector\left( {s_i } \right)} \right\| \cdot \left\| {vector\left( {s_j } \right)} \right\|}}$$

where $vector(s_i )$ represents the vector representation of abiotic stress $s_i$,$vector(s_j )$ represents the vector representation of abiotic stress $s_j$. Eventually, we obtained the semantic similarity network for abiotic stresses, which we will subsequently use to construct the final abiotic stress similarity network.

#### miRNA functional similarity

When two miRNAs share functional similarities, it is plausible that they are associated with diseases manifesting similar pathological phenomena or symptoms. Consequently, the functional similarity of miRNAs can be calculated and gauged by evaluating the similarity between the diseases with which they are associated [63, 64]. In a parallel vein, akin to miRNAs with analogous functions being linked to diseases with similar phenotypes, distinct miRNAs may also display certain functional resemblances when subjected to comparable types of abiotic stress. To quantify the functional similarity of miRNAs, we employed the methodology introduced by Wang et al. [65]. This method is often applied to measure the functional similarity between two entities in association prediction. For example, Wang et al. [66]. used this method to calculate the functional similarity network of microbes in the association prediction between microbes and releases, and combined it with other similarity networks as the feature representation of microbes.

To accurately measure the functional similarity between two miRNAs, we need also to consider the contributions from similar abiotic stress that are associated with these two miRNAs, respectively. Therefore, we initiate by defining semantic similarities between an abiotic stress and a set of abiotic stresses:

$${\text{S}}\left( {st,ST} \right) = {\mathop {\max }\limits_{1 \le i \le j}} \left( {S\left( {st,st_i } \right)} \right)$$

Here we define $st$ as an abiotic stress. $ST$ is defined as a group of abiotic stresses, that is, $ST = \{ st_1 ,st_2 ,st_3 {, }... \, st_j \}$. Subsequently, the functional similarity between miRNA $m_1$ and $m_2$ can be defined as:

$$Func\left( {m_1 ,m_2 } \right) = \frac{{\sum_{1 \le i \le m} {S\left( {st_{1i} ,ST_2 } \right) + \sum_{1 \le j \le n} {S\left( {st_{2{\text{j}}} ,ST_1 } \right)} } }}{m + n}$$

$ST_1$ represents the set of abiotic stresses associated with $m_1$, $st_{1i}$ represents an element of $ST_1$, m and n represent the amount of abiotic stresses associated with miRNA $m_1$ and miRNA $m_2$, respectively. Finally, we obtain the functional similarity network of miRNAs, and then we will use it to construct the final miRNA similarity network.

#### miRNA sequence similarity

In our experimental approach, we utilized the Chaos Game Representation (CGR) [67] technique to convert the miRNA sequence into a vector representation. CGR is an iterative sequence mapping method renowned for its capacity to faithfully restore the original sequence information of miRNA from coordinates, ensuring no loss of miRNA sequence data during the mapping process. Furthermore, it can uniquely map the miRNA sequence to a two-dimensional plane by incorporating both positional and nonlinear information. The definition of the relative position of a nucleotide in the miRNA sequence on the plane is outlined as follows:

$${\text{T}}_{\text{l}} { = 0}{\text{.5}} \times \left( {{\text{T}}_{\text{l - 1}} {\text{ + I}}_{\text{l}} } \right){\text{ l = 1,2,}}...{\text{ L}}$$

Here, L denotes the length of the miRNA sequence, while I_l_ represents the positional factor of the i-th nucleotide in the sequence, corresponding to the coordinates of the four vertices: A = (0,0), T = (1,0), C = (0,1), and G = (1,1). We initialize the starting point to be the center of the two-dimensional plane, represented as T_0_ = (0.5,0.5).

**Fig. 2:**
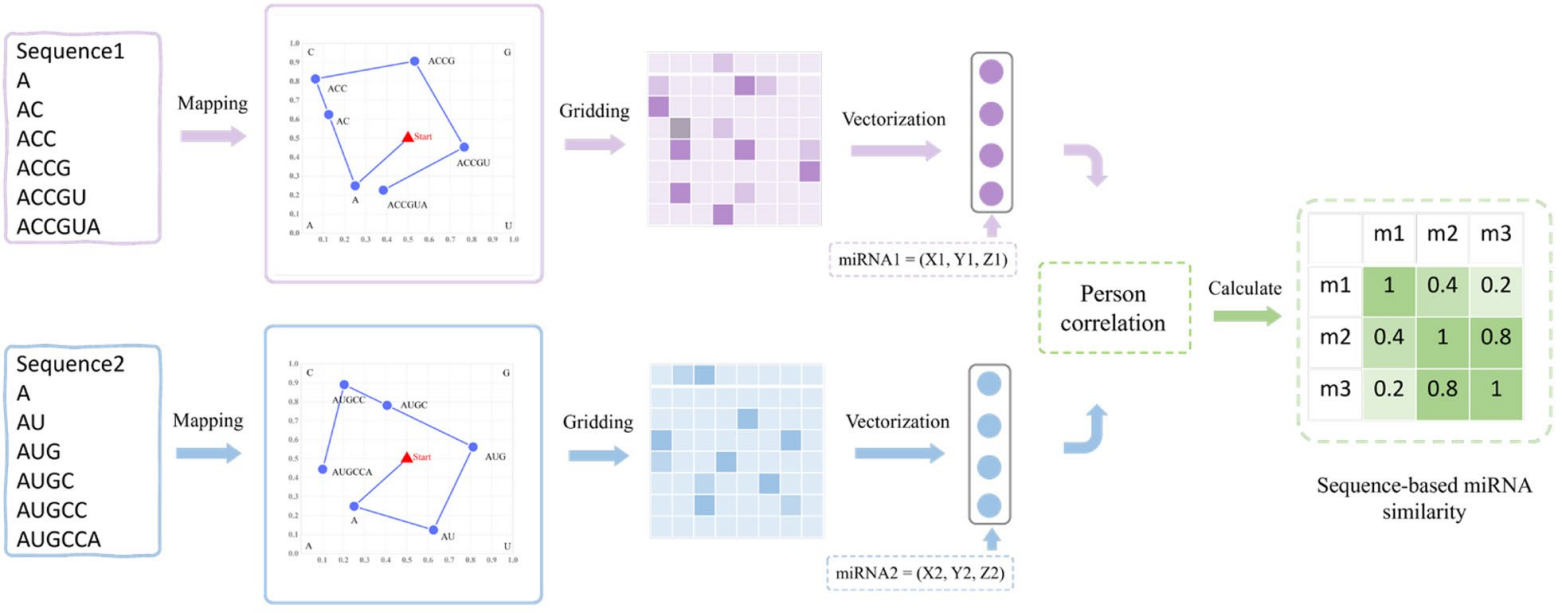
Workflow for calculating miRNA sequence similarity based on CGR technology

Figure 2 delineates the workflow for calculating miRNA sequence similarity using CGR technology. In this experiment, we employed CGR technology to encode the miRNA sequence, thereby obtaining an effective vector representation. Initially, we mapped the miRNA sequence onto a two-dimensional plane. Subsequently, we associated the relative position of each nucleotide on the two-dimensional plane with a frequency network of ${\text{N }} \times {\text{ N}}$, where N is set to 8. We then proceeded to construct the vector representation of the miRNA sequence based on the information gleaned from the frequency network. The construction method is outlined as follows:

$$vector_{\text{i}} = (X_i ,Y_i ,Z_i )$$

${\text{X}}_i$ and ${\text{Y}}_i$ represent the sum of abscissa and ordinate of all points in the i-th grid, respectively. In addition, we quantify the nonlinear information of the i-th grid using ${\text{Z}}_i$. The definition is as follows:

$$\begin{gathered} \, Z_i = \frac{{Num_i - \frac{{\sum_{j = 1}^{N \times N} {Num_j } }}{N \times N}}}{{\sqrt {{\frac{1}{N \times N}\sum_{k = 1}^{N \times N} {\left( {Num_k - \frac{{\sum_{l = 1}^{N \times N} {Num_l } }}{N \times N}} \right)^2 } }} }} \hfill \\ \hfill \\ \, Num_i = {\text{ number of points in i - th frequency network}} \hfill \\ \end{gathered}$$

Therefore, miRNA $m_i$ can be represented as the vector $vector(m_i )$. Subsequently, we use the Pearson correlation coefficient to measure the similarity between $vector(m_i )$ and $vector(m_j )$, and the calculation method is as follows:

$$Seq\left( {m_i ,m_j } \right) = \frac{{Cov\left( {vector(m_i ),vector(m_j )} \right)}}{{\sigma_{vector(m_i )} \cdot \sigma_{vector(m_j )} }}$$

where $Cov(vector(m_i ),vector(m_j ))$ represents the covariance between $vector(m_i )$ and $vector(m_j )$. $\sigma_{vector(m_i )}$ is the standard deviation of $vector(m_i )$. Finally, we obtain the sequence similarity of miRNA based on the CGR technique. Subsequently, we will use it to construct the final miRNA similarity network.

#### Gaussian interaction profile kernel similarity for miRNA and abiotic stress

When two miRNAs are similar in their functions, they are often associated with similar types of abiotic stresses. In other words, similar miRNAs may exhibit similar functional expression patterns. Here, we can evaluate the similarity of miRNAs and abiotic stresses respectively using the Gaussian interaction profile kernel (GIPK) similarity [68]. In association prediction, GIPK similarity is often used to calculate and characterize the feature information of entities based on their association information. For example, Wang et al. [66]. calculated and measured the GIPK similarity network of both microbe and disease based on microbe-disease association information, and fused this network with other types of similarity networks using a multi-source approach to fully characterize the feature information of the two entities.

Previously, we have defined the association matrix ${\text{A}} \in {\text{R}}^{{\text{m }} \times {\text{ n}}}$ between miRNA and abiotic stress, On this basis, we define $V\left( {m_i } \right)$ and $V(m_j )$, which represent the i-th row and j-th row of the association matrix ${\text{A}} \in {\text{R}}^{{\text{m }} \times {\text{ n}}}$, respectively. Then the GIPK similarity between miRNA $m_i$ and miRNA $m_j$ can be defined as follows:

$$GIPK_M \left( {m_i ,m_j } \right) = exp\left( { - \;\gamma_m \left\| {V\left( {m_i } \right)\; - \;V\left( {m_j } \right)} \right\|^2 } \right)$$

$$\gamma_m = \frac{n}{{\sum_{i = 1}^n {\left\| {V\left( {m_i } \right)} \right\|^2 } }}$$

Similarly, the GIPK similarity between abiotic stress $s_i$ and $s_j$ can be defined as:

$$GIPK_S \left( {s_i ,s_j } \right) = exp\left( { - \;\gamma_m \left\| {V\left( {s_i } \right) - \;V\left( {s_j } \right)} \right\|^2 } \right)$$

$$\gamma_s = \frac{m}{{\sum_{i = 1}^m {\left\| {V\left( {s_i } \right)} \right\|^2 } }}$$

where $V\left( {s_i } \right)$ and $V(s_j )$ respectively represent the i-th column and j-th column of the association matrix ${\text{A}} \in {\text{R}}^{{\text{m }} \times {\text{ n}}}$. Finally, we obtained the GIPK similarity network of miRNA and the GIPK similarity network of abiotic stress.

#### Integrated similarity for miRNA and abiotic stress

The final miRNA similarity network is obtained by integrating the multi-source miRNA similarity network, which comprehensively considers the sequence similarity network, the functional similarity network and the GIPK similarity network. The calculation formula of the integrated similarity network is as follows:

$$MS\left( {m_i ,m_j } \right) = \frac{{\alpha_1 Seq\left( {m_i ,m_j } \right) + \alpha_2 Func\left( {m_i ,m_j } \right) + \alpha_3 GIPK_M \left( {m_i ,m_j } \right)}}{\alpha_1 + \alpha_2 + \alpha_3 }$$

where $Seq\left( {m_i ,m_j } \right),Func\left( {m_i ,m_j } \right)$ and $GIPK_M \left( {m_i ,m_j } \right)$ represent miRNA sequence similarity network, miRNA functional similarity network and miRNA GIPK similarity network, respectively. We use $\alpha_i \left( {i \in 1,2,3} \right)$ to measure the contribution of different similarities, and here we set it to 1.

To create the final abiotic stress similarity network, we similarly integrate the multi-source similarity network of abiotic stress, which consists of the GIPK similarity network and semantic similarity networks of abiotic stress, respectively. The following is the integrated similarity network calculating method:

$$SS\left( {s_i ,s_j } \right) = \frac{{\beta_1 Sem\left( {s_i ,s_j } \right) + \beta_2 GIPK_S \left( {s_i ,s_j } \right)}}{\beta_1 + \beta_2 }$$

where $Sem\left( {s_i ,s_j } \right)$ and $GIPK_S \left( {s_i ,s_j } \right)$ respectively represent the semantic similarity network of abiotic stress and the GIPK similarity network of abiotic stress. Here the weight parameter $\beta_i \left( {i = 1,2} \right)$ is also set to 1. Finally, we obtained the integrated miRNA similarity network and the integrated abiotic stress similarity network.

### Construct miRNA-abiotic stress heterogeneous network

Using multiple similarity measurement methods, we calculated the miRNA and abiotic stress respective similarity networks from different perspectives in this study based on the multi-source feature information. These networks were then integrated to obtain the final similarity networks for miRNA and abiotic stress. In addition, we also define the association matrix ${\text{A}} \in {\text{R}}^{{\text{m }} \times {\text{ n}}}$ between miRNA and abiotic stress. In this section, we construct the miRNA-abiotic stress heterogeneous network ${\text{H}}$ based on the above three networks, and the construction method is as follows:

$${\text{H}} = \left[ \begin{gathered} {\text{ MS A}} \hfill \\ {\text{ A}}^{\text{T}} {\text{ SS}} \hfill \\ \end{gathered} \right] \in {\text{R}}^{ \, \left( {\text{m + n}} \right) \times \left( {\text{m + n}} \right)}$$

where ${\text{MS}}$ and ${\text{SS}}$ represent the integrated miRNA similarity network and the integrated abiotic stress similarity network respectively. Subsequently, we use the RWR algorithm to learn the global structure information of heterogeneous networks. Through random network walking, the RWR algorithm mimics the process of information transmission. The walker hops to the neighboring node with one probability and stays at the current node with another one at each step. The RWR technique can obtain the transition probability between nodes through numerous iterations and eventually arrive at the steady-state matrix. The steady-state matrix can be regarded as a feature vector describing the importance and interrelationship of nodes in the network. By performing the RWR algorithm on the heterogeneous network, the probability vector obtained by RWR for node *i* at step t + 1 is calculated by the following formula:

$$p_i^{t + 1} = \left( {1 - \alpha } \right)Tp_i^t + \alpha p_i$$

where $\alpha$ is the restart probability, *T* is the probability transition matrix of the heterogeneous network, $p_i$ is the n-dimensional initial feature vector, and $p_i^t$ is the n-dimensional feature vector of node *i*. With the steady-state matrix generated by the RWR algorithm, we can describe each miRNA and abiotic stress node as feature vectors, which capture their position and importance in heterogeneous networks. By doing this, we are able to obtain the feature representations of miRNA and abiotic stress, which serve as a foundation for future association prediction.

### miRNA-abiotic stress association prediction

In our research, we constructed a miRNA-abiotic stress heterogeneous network by integrating the multi-source similarity network information of miRNA and abiotic stress. The RWR algorithm was used in our study to learn the topological structure of the heterogeneous network and obtain the feature representations of miRNA and abiotic stress. Subsequently, our model will then be used to predict potential associations between miRNA and abiotic stress. Our model comprises a decoder and an encoder specifically. The encoder is built on a GIN [69] model, which can efficiently extract implicit topological information from graphs and learn an efficient representation of their structure. The feature vector of a node is iteratively updated and calculated using the nearby nodes' feature vectors, according to the neighbor aggregation approach. Additionally, GIN introduces a Multi-Layer Perceptron (MLP) to learn and model an injective function for aggregating features. The feature vector of a node at the k-th layer in GIN can be represented as:

$$h_v^{(k)} = MLP^{(k)} \left( {\left( {1 + \varepsilon^{(k)} } \right) \cdot h_v^{(k - 1)} + \sum_{u \in N(v)} {h_u^{(k - 1)} } } \right)$$

where $h_v^{(k - 1)}$ is the feature vector of node v at the (k–1)-th layer, and the feature vector of nodes at the 0-th layer is the input to our model, that is, the heterogeneous network. $N(v)$ is the set of neighborhoods of node v. In addition, if there is any difference between the feature vector of a node or neighbors of a node, we introduce a learnable parameter $\varepsilon$ to ensure that the feature vector of the node is also different. In addition, $MLP^{(k)}$ is a Multi-Layer Perceptron, which can learn the unique mapping from $\left( {1 + \varepsilon^{(k)} } \right) \cdot h_v^{(k - 1)} + \sum_{u \in N(v)} {h_u^{(k - 1)} }$ to $h_v^{(k)}$.

After iteratively updating the feature vectors of nodes in the encoder, we use a decoder to reconstruct the association matrix $\text{A}^{\prime}$ between miRNA and abiotic stress. Our decoder is defined as follows:

$$\text{A}^{\prime} = \sigma \left( {Z \cdot Z^T } \right)$$

where $\sigma$ is a nonlinear activation function, and in this case, we use the sigmoid function. Z is the output of our encoder. Our model's loss function, which calculates the difference between the predicted value and the actual value, is the cross-entropy function. The following defines the loss function:

$$L = - \frac{1}{N}\sum {y\log y^{\prime}} + (1 - y)\log (1 - y^{\prime})$$

where $y$ represents the value of an element in the association matrix ${\text{A}} \in {\text{R}}^{{\text{m }} \times {\text{ n}}}$, that is, the true value. $y^{\prime}$ represents the value of an element at the corresponding position in the reconstructed association matrix $\text{A}^{\prime}$, that is, the predicted value. Then, we use the Adam optimizer to minimize the loss function. After that, we can obtain potential associations between miRNA and abiotic stress based on the reconstructed association matrix generated by our model.

## Results and discussion

### Experimental setup and evaluation metrics

We used the K-fold cross-validation method in our experiment to assess the model's performance. In K-fold cross-validation, all known miRNA-abiotic stress association data are randomly divided into K equal subsets, one of which is utilized as the test set and the remaining K-1 subsets as the training set. The average of the K test results is used as the evaluation result after this process is repeated K times with a different subset being used each time as the test set. Here, the K value is set to 5. The benefit of using cross-validation to evaluate a model is that it can better evaluate the generalization ability of the model and provide an assessment of model stability.

Since AUPR and AUC can indicate the performance of the model at various thresholds, they were utilized as the primary evaluation metrics in this work. Additionally, we also used other threshold-based evaluation metrics such as F1 score, accuracy, recall and so on. The following are the relevant mathematical formulas:

$${\text{Precision = }}\frac{{{\text{TP}}}}{{{\text{TP}} + {\text{FP}}}}$$

$${\text{Recall = }}\frac{{{\text{TP}}}}{{{\text{TP}} + {\text{FN}}}}$$

$${\text{F1\_score = }}\frac{{2{\text{TP}}}}{{2{\text{TP}} + {\text{FN}} + {\text{FP}}}}$$

where TP, FP, TN, and FN represent true positives, false positives, true negatives, and false negatives, respectively. AUC refers to the area under the curve of the Receiver Operating Characteristic (ROC) curve, which quantitatively reflects the model's performance measured based on the ROC curve. The abscissa of the ROC curve represents the False Positive Rate (FPR), and the ordinate represents the True Positive Rate (TPR). TPR and FPR are calculated as follows:

$${\text{TPR}} = \frac{{{\text{TP}}}}{{{\text{TP}} + {\text{FN}}}}$$

$${\text{FPR}} = \frac{{{\text{FP}}}}{{{\text{FP}} + {\text{TN}}}}$$

The abscissa of the PR curve represents the model's recall, and the ordinate represents the model's precision. AUPR stands for the area under the PR curve. The larger the area under the PR curve, the better the performance of the model. In addition, our model has several important hyperparameters, such as the initial learning rate *lr*, the node embedding dimension of encoder layer 1 *hidden1*, the node embedding dimension of encoder layer 2 *hidden2*, and the dropout rate of nodes *dropout*. We explored various iterations of these parameters and carried out numerous experimental confirmations. We selected the optimal parameter combination as lr = 0.001, hidden1 = 256, hidden2 = 128, and dropout = 0. The details of parameter adjustment are shown in Table 3.

**Table 3:** The details of parameter adjustment

Parameter	Description	Search scope	Best
Learning-rate	The initial learning rate of optimizer	{0.001,0.002,0.005,0.01,0.02,0.05}	0.001
Hidden1	The embedding dimension of layer 1	{16,32,64,128,256}	256
Hidden2	The embedding dimension of layer 2	{16,32,64,128,256}	128
Dropout	The dropout rate of nodes	{0,0.2,0.4,0.5}	0

### Performance comparison with different prediction methods

**Fig. 3:**
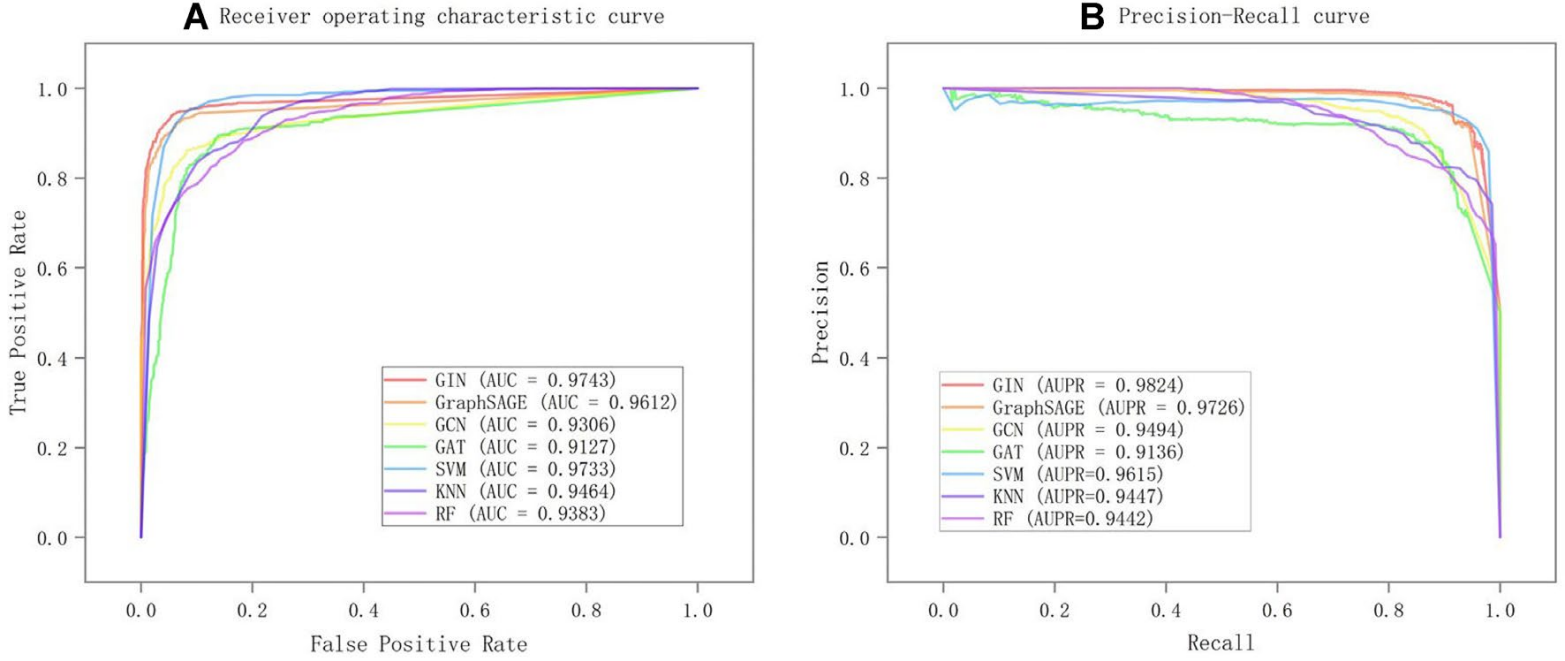
Performance of different models in predicting miRNA-abiotic stress associations (**a**) Receiver operating characteristic (ROC) curves under fivefold cross-validation (**b**) Precision-recall (PR) curves under fivefold cross-validation

In order to assess the performance of our model in predicting potential miRNA-abiotic stress associations, we first compared it to several common traditional machine learning models. Since graph structure data cannot be utilized directly to train machine learning models, we also offer machine learning models based on multi-source similarity network fusion to guarantee the consistency of training data and the rationality of comparison. Specifically, we performed the following processing: we concatenated the miRNA similarity network MS with the abiotic stress similarity network SS to form the feature vector of miRNA-abiotic stress pairs. For example, we concatenated the i-th row of the miRNA similarity network MS with the j-th row of the abiotic stress similarity network SS to form the feature vector for the miRNA $m_i$ and abiotic stress $s_j$ pair. These feature vectors are the inputs for the machine learning model, and the five-fold cross-validation is used to assess the model's performance. All parameters of the models have been adjusted to the optimal level, and the tuning details are shown in Table 4. Table 5 and Fig. 3 present the findings.The bold value is the maximum value of the column. SVM performed the best in machine learning, with improvements of 1.73% and 3.5% in AUPR and AUC compared to RF, respectively. Compared to KNN, it has increased by 1.68% and 2.68%, respectively. This indicates that SVM has the best performance and robustness among all machine learning models. Our approach outperforms SVM, the top-performing classical machine learning model, by 2.09% in terms of AUPR. Our model performs similarly to SVM in terms of AUC, with a 0.1% improvement. Our model surpasses SVM in terms of F1 score and precision by 1.67% and 4.03%, respectively, despite SVM having a 0.73% slightly greater recall than our model. This suggests that our model can maintain a high recall while achieving higher precision and F1 score. As a result, we think that our model performs better than SVM. Additionally, our approach also has outperformed more than conventional machine learning models in terms of various evaluation indicators. We believe that our model performs better in predicting potential miRNA-abiotic stress associations than conventional machine learning models when all of these aspects are taken into account.

**Table 4:** The details of parameter adjustment

Parameter	Search Scope	Best	Parameter	Search Scope	Best
	GraphSAGE			GAT	
Learning-rate	{0.001,0.002,0.005,0.01,0.02,0.05}	0.001	Learning-rate	{0.001,0.002,0.005,0.01,0.02,0.05}	0.001
Hidden1	{16,32,64,128,256}	256	Hidden1	{16,32,64,128,256}	256
Hidden2	{16,32,64,128,256}	128	Hidden2	{16,32,64,128,256}	128
Dropout	{0,0.1, 0.2,0.4,0.5}	0	Dropout	{0,0.1, 0.2,0.4,0.5}	0.1
Num_neighbors	{1,2,4,5,6,8,10}	5	Num_heads	{1,2,3,4,5,6,7,8,9,10}	2

Parameter	Search scope	Best	Parameter	Search scope	Best
	GCN			SVM	
Learning-rate	{0.001,0.002,0.005,0.01,0.02,0.05}	0.001	Kernel	{'linear', 'poly', 'rbf', 'sigmoid'}	Rbf
Hidden1	{16,32,64,128,256}	256	C	Range(50,150)	100
Hidden2	{16,32,64,128,256}	128	Gamma	{'scale', 'auto'}	Scale
dropout	{0,0.1,0.2,0.4,0.5}	0			

Parameter	Search scope	Best	Parameter	Search scope	Best
	KNN			RF	
n_neighbors	Range(1,50)	8	n_estimators	Range(150,250)	220
Weights	{'uniform', 'distance'}	Distance	Criterion	{"gini", "entropy"}	Gini
Algorithm	{'auto', 'ball_tree', 'kd_tree', 'brute'}	Auto

**Table 5:** Performance of different models in predicting miRNA-abiotic stress associations under five-fold cross-validation

Model	AUPR	AUC	F1	ACC	RE	SPE	PRE
GIN	**0.9824**	**0.9743**	**0.9495**	**0.9499**	0.9453	**0.9544**	**0.9545**
GraphSAGE	0.9726	0.9612	0.9348	0.9347	0.9368	0.9325	0.9334
GCN	0.9494	0.9306	0.8882	0.8913	0.8639	0.9186	0.9143
GAT	0.9136	0.9127	0.8843	0.8837	0.8870	0.8803	0.8822
SVM	0.9615	0.9733	0.9328	0.9320	**0.9526**	0.9117	0.9142
KNN	0.9447	0.9464	0.8652	0.8548	0.9364	0.7751	0.8052
RF	0.9442	0.9383	0.8478	0.8478	0.8568	0.8376	0.8400

F1, F1 score; ACC, accuracy; RE, recall; SPE, specificity;PRE, precision, Bold indicates the maximum value of the column

In order to further evaluate the performance of our model, we compare it with several common graph neural network models at present. Specifically, we choose GraphSAGE [70], GCN [71], and GAT [72] as the encoders, respectively, of our model and utilize the same decoder as our model to compare the performance of several graph neural network models through fivefold cross-validation on the same dataset. Table 5 and Fig. 3 present the findings. Our model outperformed GraphSAGE, which has the best performance among other graph neural networks, in terms of the AUPR and AUC indices, respectively, by 0.98% and 1.31%. In terms of F1 score, it improved by 1.47% compared to GraphSAGE. In addition, our model achieved improvements of 2.19% and 2.11% in specificity and precision, respectively. This indicates that our model can more accurately identify positive class samples and reduce misjudgment of negative class samples, and thus demonstrate the high robustness of our model. Our model improved by 3.3% and 4.37% when compared to GCN. Although GAT and GCN both produced comparable outcomes, neither model outperformed ours. Additionally, our model performs better than commonly used graph neural network models in various evaluation measures. This suggests that our model performs best at identifying potential associations between miRNA and abiotic stress.

Learning graph-structured data, such as social networks and financial networks, primarily involves learning effective representations of the graph structure. Graph Neural Networks (GNNs) are an effective framework for graph representation learning. GNN follows the neighborhood aggregation scheme, where the representation vectors of nodes are calculated by recursively aggregating and transforming the representation vectors of their neighbors. The Weisfeiler-Lehman test (WL test) [73], like GNN, aggregates the feature vectors of a particular node's neighbors to iteratively update the node's feature representation. The WL test is a reliable test with good computing efficiency. The key reason why the WL test is so powerful is its injective aggregation update mode, which can map different node neighborhoods to different feature vectors. Xu et al. [69] demonstrate that GNN is at most as powerful as the WL test in distinguishing graph structures, and further point out that if a GNN and WL test are equivalent in distinguishing or representing graph structures, then it is required that the aggregation scheme of GNN is highly expressive and can model injective functions. In the GIN model, a Multi-Layer Perceptron (MLP) is used to learn and model an injective function that combines neighbor features by the universal approximation theorem. This enables GIN as effective at identifying and resembling graph structures as WL tests in comparison to other graph neural network models that simply employ simple mean aggregation or sum aggregation. Our experimental findings also show that GIN performs better than other graph neural networks as the encoder for predicting potential miRNA-abiotic stress associations. This also explains why our model outperforms previous graph neural networks at predicting possible associations between miRNA and abiotic stress.

### Ablation experiment

#### Analysis of ablation studies of similarity network

In this research, we combined data from multiple similarity networks of miRNA and abiotic stress, creating a heterogeneous network of miRNA and abiotic stress as the model's input. In this section, we perform ablation experiments to confirm the contribution of different similarity networks to our model. Specifically, we constructed five sets of experiments, one by one deleting the similarity networks of "Seq", "Func", "GIPKm", "Sem", and "GIPKs" to verify the contribution and importance of different similarity networks. We built heterogeneous networks as input for our model based on these five various combination strategies, and we used five-fold cross-validation to assess how the performance of our model was affected by the various similarity networks. Each group received the identical training methods as those outlined in "Experimental setup and evaluation metrics" section.

Table 6 presents the experimental results.The bold value is the maximum value of the column. We can see that when removing the miRNA sequence similarity network, the performance of the model decreased by 1.65% and 1.13% on the AUPR and AUC metrics, respectively. Similarly, when removing the miRNA functional similarity network, the performance of the model decreased by 1.08% and 1.65% on AUPR and AUC metrics, indicating that both the miRNA sequence similarity network and functional similarity network can improve the performance of our model. When removing abiotic stress semantic similarity networks, the model performance decreased by 0.97% and 1.45% on the AUPR and AUC indicators, respectively. In addition, we can observe that in the two experiments that removed GIPK similarity, the performance of the model decreased the most, reaching 2%. This indicates that the GIPK similarity network can significantly improve the performance of our model in predicting miRNA-abiotic stress associations. In addition, the model performed the best when integrating five similarity networks.

**Table 6:** Results of similarity network ablation experiment using five-fold cross-validation

Network					AUPR	AUC	F1	ACC	RE	SPE	PRE
Seq	Func	GIPK_m_	Sem	GIPK_s_							
✗	✓	✓	✓	✓	0.9659	0.9603	0.9279	0.9277	0.9301	0.9253	0.9257
✓	✗	✓	✓	✓	0.9716	0.9578	0.9269	0.9280	0.9125	0.9435	0.9434
✓	✓	✗	✓	✓	0.9691	0.9572	0.9247	0.9256	0.9149	0.9362	0.9352
✓	✓	✓	✗	✓	0.9727	0.9598	0.9329	0.9338	0.9228	0.9447	0.9435
✓	✓	✓	✓	✗	0.9670	0.9508	0.9252	0.9265	0.9064	0.9466	0.9461
✓	✓	✓	✓	✓	**0.9824**	**0.9743**	**0.9495**	**0.9499**	**0.9453**	**0.9544**	**0.9545**

Bold indicates the maximum value of the column

In conclusion, the results conclusion, the results of the ablation experiment demonstrate that each similarity network can significantly enhance the performance of our model, demonstrating the efficacy of the method of integrating multi-source similarity networks. Different similarity networks contribute to the performance of our model to varying degrees, with the GIPK similarity network making the largest contribution to the enhancement of performance.

#### Analysis of ablation studies of known associations

The number of known miRNA-abiotic stress associations is indeed an important factor that can significantly impact the predictive performance of a model. To delve deeper into the influence of the number of associations on our model's performance, we conducted an ablation experiment, randomly selecting varying numbers of association data from the known miRNA-abiotic stress association dataset according to certain proportions. The goal was to evaluate how different numbers of associations affect the model's performance. Specifically, we started with a total of 823 known pairs of miRNA-abiotic stress associations and constructed datasets with 600, 400, and 200 pairs of associations by randomly reducing the number of associations by a quarter each time. Additionally, a dataset with 300 pairs of associations was added for a more comprehensive evaluation. The impact of association numbers on our model's performance was assessed using five-fold cross-validation.

The experimental results, as presented in Table 7, demonstrate a gradual decline in the model's performance as the number of known miRNA-abiotic stress associations decreases.The bold value is the maximum value of the column. Even when there were only 200 associations, the AUPR and AUC metrics of our model reached 0.9443 and 0.8881, respectively, outperforming the best-performing GraphSAGE model among other graph neural network models. This indicates that our model can still achieve remarkable performance even with a small number of associations. Furthermore, when only 100 pairs of associations were added, our model exhibited a 1.61% increase in AUPR and a 3.27% increase in AUC, while GraphSAGE saw only a 0.09% increase in AUPR and a 0.92% increase in AUC. This suggests that our model can make more efficient use of associations to predict potential miRNA-abiotic stress associations.

**Table 7:** Results of the ablation experiment with the associated number under five-fold cross-validation

Models	Known associations	AUPR	AUC
GIN	200	0.9443	0.8881
	300	0.9604	0.9208
	400	0.9665	0.9324
	600	0.9716	0.9548
	823	**0.9824**	**0.9743**
	200	0.9415	0.8852
	300	0.9424	0.8944
GraphSAGE	400	0.9504	0.9043
	600	0.9606	0.9356
	823	**0.9726**	**0.9612**
	200	0.8933	0.8138
	300	0.9010	0.8327
GCN	400	0.9135	0.8503
	600	0.9227	0.8839
	823	**0.9494**	**0.9306**
	200	0.8832	0.8256
	300	0.8919	0.8345
GAT	400	0.8910	0.8675
	600	0.9009	0.8937
	823	**0.9136**	**0.9127**

Bold indicates the maximum value of the column

In summary, the ablation experiments clearly illustrate that the number of known miRNA-abiotic stress associations has a significant impact on our model's performance. Reducing the number of known associations results in a decrease in predictive performance. However, even when associations are limited, our model remains highly effective. Additionally, our model demonstrates a more substantial performance improvement with the addition of a small number of associations, highlighting its ability to effectively leverage associations for prediction.

### Performance analysis of the model under different number of encoder layers

**Fig. 4:**
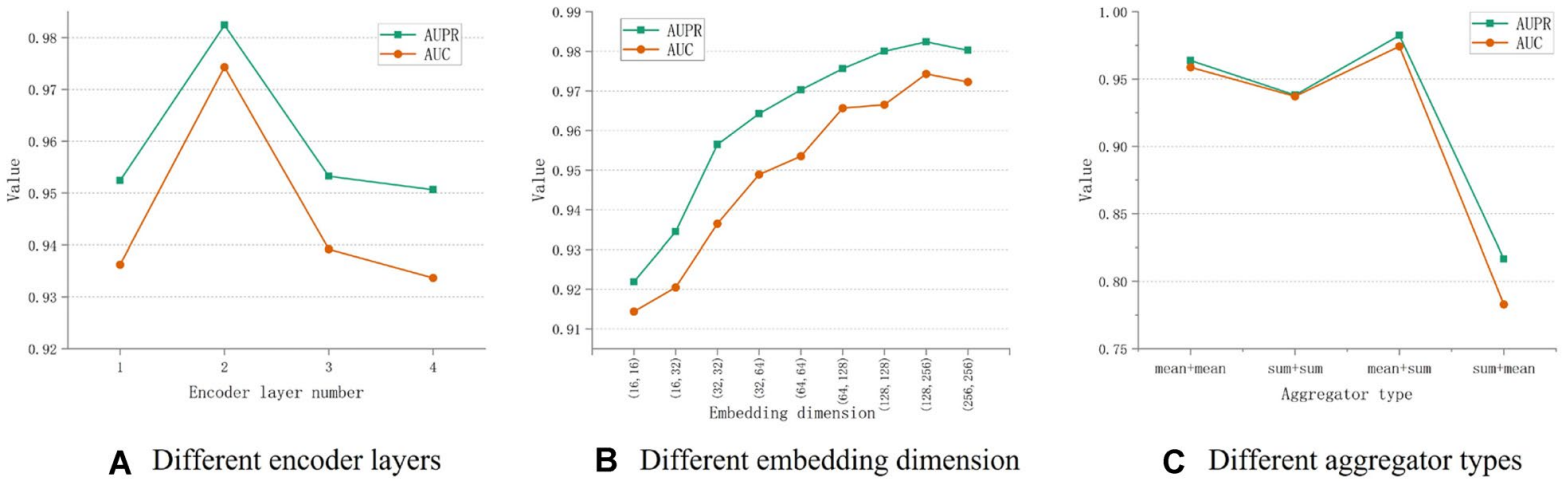
Performance of the model with different experimental parameters

The number of encoder layers in the model plays a crucial role in determining its predictive performance. In our study, we constructed the encoder of our model based on GIN, which updates the vector representations of nodes by iteratively aggregating neighbor information. The number of GIN layers in the encoder can have a significant impact on how effectively node information is aggregated, which, in turn, affects the model's predictive performance. To evaluate the impact of different encoder layer numbers, we conducted experiments and presented the results in Fig. 4A using fivefold cross-validation.

The findings indicate that the model performs best in terms of AUPR and AUC metrics when the number of encoder layers is set to 2. When the number of encoder layers is 1, the model's performance is lower than with 2 layers. This suggests that a smaller number of encoder layers may not efficiently capture the vector representation of nodes in the network.

Surprisingly, when the number of encoder layers exceeds 2, the model's performance rapidly declines. This can be attributed to the structure of the miRNA-abiotic stress interaction network, where information related to specific miRNAs and abiotic stresses is primarily found in limited node neighborhoods. Information in close neighborhoods typically represents data directly relevant to the node, whereas information from distant neighborhoods may include irrelevant or misleading data. As a result, with more encoder layers, the model aggregates information from distant neighborhoods, leading to misleading information in the feature vectors of nodes and subsequent degradation in predictive performance.

It's important to note that in our initial experiments, we encountered training difficulties when the number of encoder layers exceeded 4. In these cases, both AUPR and AUC metrics sharply dropped to 0.5. Consequently, we did not consider the scenario with more than 4 encoder layers.

In summary, the number of encoder layers in our model significantly impacts its predictive performance. A moderate number of layers, such as 2, appears to be the most effective choice, as it allows the model to capture essential information while avoiding the introduction of misleading data from distant neighborhoods.

### Performance analysis of the model under different embedding dimensions

The node embedding dimension at each layer of the encoder is a critical factor influencing the predictive performance of our model. In this section, we delved into understanding the impact of varying node embedding dimensions on the predictive performance of our model. Specifically, we explored different combinations of embedding dimensions for the two layers of the encoder, ranging from 16 to 256. we conducted a total of nine experiments to assess the predictive performance based on these combinations.

The findings indicated that the best predictive performance, in terms of both AUPR and AUC metrics, was achieved when encoder layer 1 had an embedding dimension of 128 and encoder layer 2 had an embedding dimension of 256. This suggests that this particular combination allowed the model to effectively learn and represent the features of nodes in the miRNA and abiotic stress interaction network, as well as their neighborhoods.

Furthermore, the results showed a gradual decline in predictive performance as the embedding dimensions in each layer decreased. This decline can be attributed to the encoder's inability to effectively learn the feature information of the nodes in the miRNA and abiotic stress interaction network and the nodes in their neighborhood when the embedding dimensions are low.

Interestingly, the predictive performance of the model declined when the embedding dimension combination for both layers was (256, 256) compared to the combination of (128, 256). This could be due to the encoder learning redundant miRNA and abiotic stress features when the embedding dimension is excessively high, ultimately affecting the model's prediction performance.

Taking these findings into account, we choseto select the combination of encoder layer 1 having an embedding dimension of 128 and encoder layer 2 having an embedding dimension of 256, as it yielded the best predictive performance for our model.

### Performance analysis of the model under different aggregator types

In the aforementioned investigation, we systematically discussed several critical parameters concerning our encoder architecture. We have concluded that our model achieves optimal performance in predicting potential miRNA-abiotic stress associations when employing a two-layer encoder with specific configurations: the first encoder layer embedding dimension is set to 128, and the second encoder layer embedding dimension is set to 256. Furthermore, we emphasized the significance of the aggregation type within the encoder, as it significantly influences the predictive efficacy of our model by amalgamating neighboring node information. The diverse aggregation types manifest distinct aggregation effects. To elucidate this, we conducted experiments utilizing two primary aggregation types "Mean" and "Sum" and considered various combinations thereof. Consequently, we structured four experimental groups denoted as (mean, mean), (sum, sum), (mean, sum), and (sum, mean), where the first value denotes the aggregation type of the first encoder layer, and the second value denotes the aggregation type of the second encoder layer. The impact of these distinct aggregation types on the predictive performance of our model was meticulously assessed via fivefold cross-validation, the results of which are presented in Fig. 4C. Notably, our model demonstrated superior predictive performance in forecasting potential miRNA-abiotic stress associations when employing the "Mean" aggregation type for the first encoder layer and the "Sum" aggregation type for the second encoder layer. Additionally, we observed that when both layers of the encoder employed either "Mean" or "Sum" aggregation types, utilizing "Sum" for both encode layers resulted in diminished prediction performance. This observation suggests that the "Mean" aggregation type facilitates a more effective acquisition of node vector representations within the interaction network between miRNA and abiotic stress. Conversely, adopting the "Sum" aggregation type for both encode layers may fail to capture crucial information about the miRNA-abiotic stress interaction network. We further noted that the predictive performance of the model was least favorable when the first encoder layer employed the "Sum" aggregation type while the second encoder layer employed the "Mean" aggregation type. One plausible explanation for this is that the first-layer neighborhood of a node contains directly relevant information to the node, and an encoder with the "Sum" aggregation type may inadequately discern the most pertinent information within this neighborhood. On the other hand, when the second encoder layer adopts the "Mean" aggregation type, the node features gleaned by the first encoder layer are averaged with the information from nodes in the second-layer neighborhood. However, this averaging process may lead to the loss of intricate details in node features learned by encoder layer 1, ultimately resulting in suboptimal predictive performance of the model. Consequently, our conclusive determination advocates the adoption of the "Mean" aggregation type for the first encoder layer and the "Sum" aggregation type for the second encoder layer.

## Case study

**Fig. 5:**
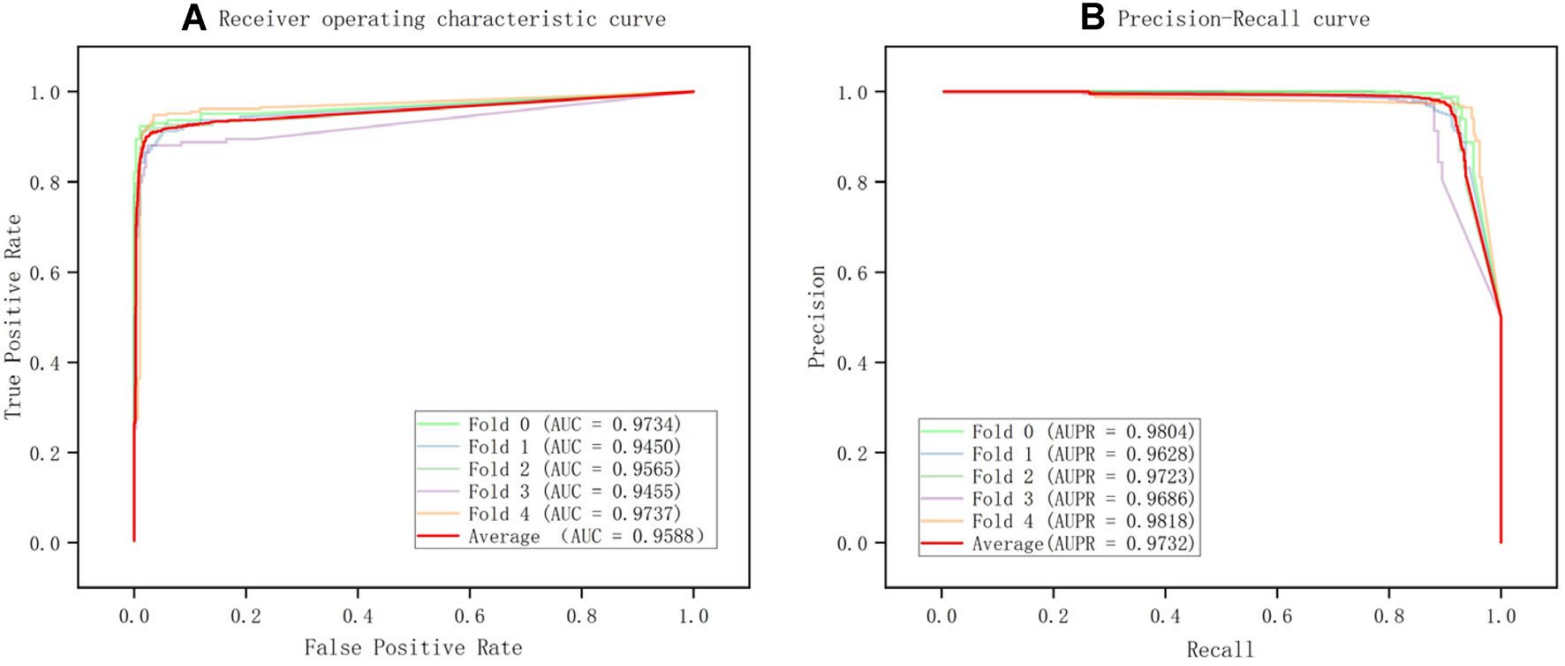
Performance of our model using PAS-MIR dataset under five-fold cross-validation (**a**) Receiver operating characteristic (ROC) curves (**b**) Precision-recall (PR) curves under five-fold cross-validation

We collected the additional 714 pairs of miRNA-abiotic stress associations from the PAS-MIR database [47] and used the same data preprocessing method as in the response to Comment d. Subsequently, we further validated the performance of our model from two aspects. Firstly, we conducted five-fold cross-validation using this dataset to evaluate the performance of our model and the results are shown in Table 8 and Fig. 5. The AUPR metric and AUC metric reached 0.9732 and 0.9588, respectively. This indicates that our model has good performance in predicting the miRNA-abiotic stress association.

**Table 8:** Performance of our model in predicting miRNA-abiotic stress associations under five-fold cross-validation

Fold	AUPR	AUC	F1	ACC	RE	SPE	PRE
0	0.9804	0.9734	0.9507	0.9509	0.9474	0.9544	0.9541
1	0.9628	0.9450	0.9183	0.9211	0.8877	0.9544	0.9511
2	0.9723	0.9565	0.9274	0.9316	0.8737	0.9895	0.9881
3	0.9686	0.9455	0.9165	0.9211	0.8667	0.9754	0.9724
4	0.9818	0.9737	0.9522	0.9531	0.9340	0.9722	0.9711
Average	0.9732	0.9588	0.9330	0.9355	0.9019	0.9692	0.9674

On the other hand, we compiled the top 10 miRNAs associated with common abiotic stresses (cold, drought and heat) based on the predicted results of our model, as shown in Table 9.

**Table 9:** The top 10 results predicted in our model based on the dataset

Rank	miRNA	Evidence	Rank	miRNA	Evidence
Cold					
1	osa-miR156k	PMID: 23,994,683	6	osa-miR171a	PMID: 23,994,683
2	osa-miR168b	PMID: 23,994,683	7	osa-miR167b	PMID: 23,994,683
3	osa-miR166k	PMID: 23,994,683	8	tae-miR159	PMID: 24,735,552
4	osa-miR166m	PMID: 23,994,683	9	osa-miR167a	PMID: 23,994,683
5	osa-miR167	DOI:10.1111/jac.12114	10	osa-miR167i	Unknown
Drought					
1	gma-miR166b	PMID: 22,112,171	6	ppt-miR1054	PMID: 21,641,561
2	pvu-miR159	PMID: 20,591,534	7	ppt-miR1028a	PMID: 21,641,561
3	gma-miR169d	PMID: 22,112,171	8	gma-miR397a	PMID: 21,663,675
4	gma-miR394a	PMID: 22,112,171	9	ath-miR395c	PMID: 20,839,006
5	ath-miR319c	PMID: 15,258,262	10	ath-miR168a	PMID: 22,938,544
Heat					
1	ptc-miR1444	PMID: 18,363,789	6	ptc-miR482	PMID: 18,363,789
2	ptc-miR827	PMID: 18,363,789	7	ptc-miR1450	PMID: 18,363,789
3	ptc-miR530a	PMID: 18,363,789	8	ath-miR156	PMID: 24,769,482
4	tae-miR159	PMID: 20,573,268	9	tae-miR160	PMID: 20,573,268
5	ath-miR156h	PMID: 24,769,482	10	ptc-miR1447	PMID: 18,363,789
Copper (Cu) deficiency					
1	ptc-miR398	PMID: 21,941,002	6	ath-miR398a	PMID: 20,400,527
2	ath-miR408	PMID: 18,408,011	7	bna-miR399	PMID: 20,388,194
3	ath-miR398b	PMID: 19,122,104	8	ath-miR857	PMID: 18,408,011
4	ath-miR397	PMID: 18,408,011	9	bna-miR2111	PMID: 20,388,194
5	ath-miR398c	PMID: 19,122,104	10	bna-miR397	PMID: 20,388,194

Cold stress is one of the common abiotic stresses that affect temperate seed crops and may have serious impacts on plant development and growth, including reduced yield and death [74, 75]. When studying candidate miRNAs related to cold stress, it was discovered that the top 9 miRNAs have been experimentally confirmed to exhibit changes in expression under cold stress. For example, based on high-throughput sequencing results, researchers conducted qRT-PCR validation and observed that the expression level of osa-miR167, ranked fifth, was up-regulated under cold stress [76]. Furthermore, RT-qPCR results demonstrated that the eighth-ranked tae-miR159 was significantly up-regulated under cold stress. The results of the Northern Blot analysis further confirmed the significant up-regulation of this miRNA at different time points, with peak expression observed at 6 hpt and 48 hpt, respectively [77].

Drought stress is a significant environmental factor that impacts crop yield through changes in plant development, metabolism, and gene expression. The top 10 miRNAs among candidate miRNAs related to drought stress have been experimentally confirmed to exhibit changes in expression under drought stress. For instance, researchers have discovered that the first and third-ranked gma-miR166b and gma-miR169d were significantly up-regulated in expression under drought stress by combining deep sequencing technology with in-depth bioinformatics analysis [78]. The fifth-ranked ath-miR319c has been validated to be down-regulated in Arabidopsis through Northern Blot analysis [18], whereas the ninth-ranked ath-miR395c is up-regulated [18].

Heat, often combined with drought stress, leads to yield losses and reduced food quality [79]. Among the top 10 miRNAs predicted by our model to be related to high temperature, all of them have been validated by relevant literature. This shows that our model can accurately predict miRNAs associated with heat stress. For example, Lu et al. [80] used northern blot analysis with probes containing complementary sequences to analyze expression levels in the leaves, phloem, and developing xylem of Populus. It was confirmed that the expression of ptc-miR1444, ptc-miR827, ptc-miR530a, ptc-miR482, ptc-miR1450 and ptc-miR1447 ranked 1, 2, 3, 6, 7, and 10 were significantly down-regulated. Among them, ptc-miR1447 and ptc-miR827 showed higher response to heat stress.

Copper is an essential mineral for the healthy growth and development of plants. Among the candidate miRNAs associated with Copper (Cu) deficiency stress, ptc-miR398 is ranked first, and studies have confirmed that its expression level is down-regulated with increasing Cu concentration in the culture medium [81]. Additionally, researchers conducted RNA blot analysis and observed that the expression levels of ath-miR398b, ath-miR398c, and ath-miR398a, ranked third, fifth, and sixth, respectively, were up-regulated [82].

## Conclusion

An increasing body of research underscores the pivotal role that microRNA (miRNA) plays in orchestrating plant responses to diverse abiotic stresses. In light of this, our study proposes a method predicated on multi-source similarity network fusion and graph autoencoder to predict potential associations between miRNA and abiotic stress. Initially, we constructed a miRNA-abiotic stress association matrix founded on the miRNA-abiotic stress association data. Subsequently, we comprehensively accounted for multi-source feature information concerning miRNA and abiotic stress, calculating similarity networks for miRNA and abiotic stress from diverse perspectives employing multiple similarity metrics. These calculated multi-source similarity networks were integrated. Following integration, we fused the resultant miRNA similarity network, the integrated abiotic stress similarity network, and the miRNA-abiotic stress association matrix to construct a heterogeneous network capturing miRNA-abiotic stress associations. We applied the RWR to learn node representations within the network, thus obtaining feature representations for miRNA and abiotic stress. Ultimately, our model facilitated the prediction of potential miRNA-abiotic stress associations. The model comprises an encoder and a decoder. The encoder is constructed on the GIN model, known for efficiently learning representations of graph structures. The decoder, on the other hand, reconstructs the miRNA-abiotic stress association matrix based on the miRNA-abiotic stress feature vector learned by the encoder. Subsequently, potential associations between miRNA and abiotic stress are inferred based on the reconstructed association matrix generated by our model. Despite the impressive performance exhibited by our model, it is important to acknowledge its limitations. Notably, the feature information about abiotic stress has not been exhaustively explored, and the fusion of multi-source similarity networks is based on a simple weighted method. In the future, we intend to develop a novel similarity network fusion method. Additionally, we aspire to comprehensively consider the feature information of abiotic stress from diverse perspectives, encompassing a wider array of biological entities such as genes, targets, etc., to construct a heterogeneous network that encompasses a broader spectrum of entities and associations. This expansion will enhance our model's capacity to predict potential miRNA-abiotic stress associations.

## Data Availability

The association data between miRNA and abiotic stress was obtained from the public database PncStress (https://bis.zju.edu.cn/pncstress/). The association data between miRNA and abiotic stress, the processing code for multi-source features, and the specific code implemented in this study can all be found at https://github.com/Chliming/MFGNN.
